# Unveiling and validating biomarkers related to the IL-10 family in chronic sinusitis with nasal polyps: insights from transcriptomics and single-cell RNA sequencing analysis

**DOI:** 10.3389/fmolb.2024.1513951

**Published:** 2025-01-03

**Authors:** Xinghong Liu, Yi Peng, Ling Guo, Weilan Xiong, Weijiang Liao, Jiangang Fan

**Affiliations:** ^1^ Department of Otolaryngology Head and Neck Surgery, Sichuan Provincial People’s Hospital, University of Electronic Science and Technology of China, Chengdu, China; ^2^ Department of Otolaryngology Head and Neck Surgery, Chengdu Second People’s Hospital, Chengdu, China; ^3^ Department of Otolaryngology Head and Neck Surgery, Sichuan Provincial People’s Hospital, Chengdu University of Traditional Chinese Medicine, Chengdu, China

**Keywords:** IL-10 family, chronic rhinosinusitis with nasal polyps, biomarkers, bioinformatics, therapeutic targets

## Abstract

**Introduction:**

Extensive efforts have been made to explore members of the IL-10 family as potential therapeutic strategies for various diseases; however, their biological role in chronic rhinosinusitis with nasal polyps (CRSwNP) remains underexplored.

**Methods:**

Gene expression datasets GSE136825, GSE179265, and GSE196169 were retrieved from the Gene Expression Omnibus (GEO) for analysis. Candidate genes were identified by intersecting differentially expressed genes (DEGs) between the CRSwNP and control groups (DEGsall) with those between the high- and low-score groups within the CRSwNP cohort (DEGsNP). Biomarker selection was performed using the Least Absolute Shrinkage and Selection Operator (LASSO), Support Vector Machine Recursive Feature Elimination (SVM-RFE), and the Boruta algorithm. Further refinement of biomarkers was carried out using receiver operating characteristic (ROC) analysis, with genes demonstrating an area under the curve (AUC) greater than 0.7 being considered significant. Genes exhibiting consistent expression trends and significant differences across both GSE136825 and GSE179265 were selected as potential biomarkers. Cell-type annotation was performed on GSE196169, and the expression profiles of the biomarkers across various cell types were analyzed. A competing endogenous RNA (ceRNA) network and a biomarker-drug interaction network were also established. Additionally, the mRNALocater database was utilized to determine the cellular localization of the identified biomarkers.

**Results:**

The intersection of 1817 DEGsall and 24 DEGsNP yielded 15 candidate genes. Further filtering through LASSO, SVM-RFE, and Boruta led to the identification of seven candidate biomarkers: PRB3, KRT16, MUC6, SPAG4, FGFBP1, NR4A1, and GSTA2. Six of these genes demonstrated strong diagnostic performance in GSE179265, while four biomarkers, showing both significant differences and consistent expression trends, were validated in both GSE179265 and GSE136825. Single-cell sequencing analysis of GSE196169 revealed seven distinct cell types, including endothelial cells, with the biomarkers predominantly expressed in epithelial cells. The ceRNA network comprised nine nodes and eleven edges, with only FGFBP1 exhibiting a complete lncRNA-miRNA-mRNA interaction.

**Discussion:**

This study identifies several novel biomarkers and their associated drugs for CRSwNP therapy, as well as potential therapeutic targets, such as spiperone and arnenous acid, identified through molecular docking. Ultimately, this work underscores the identification of four IL-10 family-related biomarkers, providing a theoretical foundation for future clinical research in CRSwNP.

## 1 Introduction

Chronic rhinosinusitis (CRS) is a complex inflammatory disorder of the nasal sinus mucosa, characterized by symptoms such as nasal congestion, obstruction, edema, and discharge, often accompanied by facial swelling and impaired olfactory function (Almosnino and Little, 2023). The CRS phenotype is classified into chronic rhinosinusitis with nasal polyps (CRSwNP) and chronic rhinosinusitis without nasal polyps (CRSsNP) based on the presence of nasal polyps (Striz et al., 2023). Although these subtypes share overlapping symptoms, CRSwNP is associated with more severe nasal symptoms and higher symptom scores compared to CRSsNP (Lee et al., 2021). CRSwNP is primarily driven by a Th2-skewed inflammatory response and eosinophil infiltration. Its pathogenesis involves epithelial damage, disruption of mucosal barriers, and increased exposure to pathogens, antigens, and particles, which trigger both innate and adaptive immune responses in subepithelial tissues (Vanderhaegen et al., 2022). Patients with CRSwNP frequently experience severe symptoms, high recurrence rates, and a greater risk of comorbid asthma, significantly affecting their quality of life and work productivity (Danielides et al., 2022). This highlights the urgent need to identify novel biomarkers for early diagnosis and therapeutic intervention in CRSwNP, alongside the development of new treatment strategies.

Emerging evidence suggests that the pathogenesis of CRSwNP involves not only impaired nasal epithelial barrier function, dysregulated immune responses, and microbial colonization but also mechanisms linked to autoimmunity (Huang and Xu, 2023). First identified in 1989, IL-10 was initially characterized as a cytokine produced by Th2 cells (Ouyang et al., 2011). The IL-10 family of cytokines, which share structural and receptor similarities with IL-10, includes IL-19, IL-20, IL-22, IL-24, IL-26, IL-28A, IL-28B, and IL-29 (Fickenscher et al., 2002). Despite their structural resemblance, these cytokines have diverse roles in immune regulation and are secreted by a wide range of innate and adaptive immune cells, such as monocytes, B cells, T cells, NK cells, and macrophages, as well as structural cells like epithelial and endothelial cells (Ouyang et al., 2011). Their broad immunomodulatory functions have prompted numerous investigations into their therapeutic potential in autoimmune diseases, cancer, and inflammatory conditions (Ouyang and O'Garra, 2019). Within the context of CRSwNP, the IL-10 family plays multifaceted roles, influencing epithelial integrity, allergen responses, and viral or bacterial infections. Some IL-10 family cytokines have already been evaluated in clinical trials targeting airway inflammatory conditions (Xuan et al., 2022). Thus, they hold promise as potential therapeutic agents in CRSwNP.

This study leveraged publicly available CRSwNP-related datasets and applied comprehensive bioinformatics approaches to identify IL-10 family-associated biomarkers. Through rigorous screening and analysis, the study explored the involvement of these biomarkers in key biological pathways and their molecular regulatory mechanisms, including their interactions with disease-related drugs. The findings provide valuable insights into the pathogenesis and treatment of CRSwNP, offering a foundation for future research and therapeutic advancements.

## 2 Materials and methods

### 2.1 Data source

Three datasets were retrieved from the GEO database (https://www.ncbi.nlm.nih.gov/geo/). The training set, GSE136825 (GPL20301), included 42 CRSwNP tissue samples and 28 control tissue samples. The validation set, GSE179265 (GPL24676), comprised 17 CRSwNP tissue samples and 7 control tissue samples. Additionally, GSE196169 (GPL21290) contained 9 CRSwNP tissue samples. The IL-10 family genes analyzed in this study included IL-10, IL-19, IL-20, IL-22, IL-24, IL-26, IL-28A, IL-28B, and IL-29 (Xuan et al., 2022).

### 2.2 Differential expression analysis

For GSE136825, differential gene expression between CRSwNP and control samples (DEG_all) was analyzed using the DESeq2 package in R (McDermaid et al., 2019). To evaluate IL-10 family genes, the GSVA package in R (Lei et al., 2021) was used to calculate sample-specific scores. Based on the median score, CRSwNP samples were divided into high- and low-score groups. Differentially activated pathways were identified between these two groups using GSVA, while differential gene expression analysis (DEG_NP) was performed using DESeq2. The thresholds for DEG screening (both DEG_all and DEG_NP) were set at |log2 (fold change)| ≥1 and adjusted *p*-value <0.05. Volcano plots were generated with the ggplot2 package (v3.3.6) (Ren et al., 2022), and heatmaps were created using the pheatmap package (v1.0.12) (Zhang et al., 2021).

### 2.3 Enrichment analysis of candidate genes

Candidate genes were identified by intersecting DEG_all and DEG_NP. Enrichment analysis, including Gene Ontology (GO) and Kyoto Encyclopedia of Genes and Genomes (KEGG) pathways, was performed on these candidate genes using the ClusterProfiler package (v3.18.1) in R (Zhou et al., 2021).

### 2.4 Machine learning

To further identify genes with strong diagnostic potential, this study employed machine learning approaches to screen biomarkers based on candidate genes. First, the expression levels of candidate genes were analyzed using least absolute shrinkage and selection operator (LASSO) regression, implemented through the glmnet (v4.1-4) package in R (Wang et al., 2020), to identify feature genes. Next, a support vector machine-recursive feature elimination (SVM-RFE) model was applied to assess differential gene expression between groups, utilizing the e1071 package (v1.7-11) in R (Xu et al., 2021), followed by recursive elimination of non-essential features. Additionally, the Boruta algorithm was used to determine the most important features by comparing the z-values of each gene (Yue et al., 2022). Using a cutoff of 2.512, the Boruta algorithm screened for key feature genes among the candidate genes.

### 2.5 Identification and validation of biomarkers

The results from the three machine learning models were intersected to obtain candidate biomarkers. Receiver operating characteristic (ROC) curves for these biomarkers were generated using the pROC package in R (Gong et al., 2020), and the area under the curve (AUC) was calculated. Biomarkers with an AUC greater than 0.7 in the training set (GSE136825) were selected for further analysis. Subsequently, the expression levels of these candidate biomarkers were validated in GSE136825 and GSE179265. Biomarkers with significant differences and consistent expression trends across both datasets were identified as final biomarkers.

### 2.6 Establishment and assessment of a nomogram

A nomogram was constructed to assess the risk of CRSwNP based on selected biomarkers, assigning a score to each factor. The total score, calculated by summing the scores of all factors, corresponded to the predicted incidence of CRSwNP, with higher scores indicating an elevated risk. The nomogram was developed using the rms package (v6.3-0) in R (Liu et al., 2021). To evaluate its predictive accuracy, calibration curves were generated.

### 2.7 Functional enrichment analysis

To further investigate the signaling pathways and biological mechanisms associated with the identified biomarkers, correlation analysis was performed to calculate correlation coefficients between biomarkers and other genes. The correlated genes were ranked by their coefficients and subjected to Gene Set Enrichment Analysis (GSEA) using the KEGG gene set. The thresholds for GSEA were set at adjusted *p*-value <0.05 and |NES| > 1. Interactions among the biomarkers were examined using GeneMANIA, which integrated data from multiple large-scale biological datasets to identify genes involved in related pathways and processes.

### 2.8 Filtering and controlization of single-cell RNA sequencing data (scRNA-seq)

For GSE196169, data preprocessing and quality control were conducted using the PercentageFeatureSet function from the Seurat package (v5.0.1) (Hao et al., 2021). To mitigate dropout effects and filter low-quality cells and genes, specific criteria were applied: the number of expressed genes per cell was capped at 4,000, counts per cell were limited to below 4,000 (with most below 3,000), and mitochondrial genes were excluded. Cells were retained if they expressed between 200 and 4,000 genes, total counts were below 4,000, and mitochondrial gene expression was less than 3% (4,000 > nFeature_RNA >200, nCount_RNA <4,000, percent_mito <3).

### 2.9 Dimension reduction, unsupervised clustering, and visualization

Principal component analysis (PCA) was employed to reduce data dimensionality while preserving key information, with a higher principal component indicating richer differential component. Principal components were ranked by the percentage of variance explained, and those preceding the PCA inflection point were selected for further analysis. Clustering of all cells was performed using the FindNeighbors and FindClusters functions in the Seurat package (Yu et al., 2022). Nonlinear dimensionality reduction using UMAP was applied to visualize clusters, grouping cells into distinct subclasses. Annotation of these subtypes was conducted using the singleR tool and the CellMarker database. Differential gene expression analysis was conducted across different cell clusters within CRSwNP samples using the FindMarker function in GSE196169. The criteria for differential expression were set at |log2FC| ≥ 1 and *p*-value <0.05. Finally, the expression levels of the identified biomarkers were analyzed across different cell clusters within GSE196169 to elucidate their distribution and roles at the cellular level.

### 2.10 Regulatory network

To investigate the regulatory mechanisms of biomarkers in CRSwNP, StarBase and miRWalk databases were utilized to predict associated miRNAs. Subsequently, lncRNAs related to these miRNAs were predicted using StarBase and miRcode. The resulting competing endogenous RNA (ceRNA) network was visualized using Cytoscape software. Given that single nucleotide polymorphisms (SNPs) within coding regions can alter protein amino acid composition, leading to structural or functional changes and affecting gene activity, SNPs associated with miRNAs in the ceRNA network were predicted using the miRNASNP database. This enabled the construction of miRNA-SNP-biomarker combinations and interaction networks.

### 2.11 Subcellular localization

To understand the functional localization of biomarkers within cells, subcellular localization was predicted using the mRNALocater database. The sequences of the four biomarkers were obtained from NCBI (https://www.ncbi.nlm.nih.gov/). Subcellular localization predictions covered compartments such as the cytoplasm, endoplasmic reticulum, extracellular region, mitochondria, and nucleus. The expression of biomarkers at the single-cell level was further analyzed to elucidate their functional roles.

### 2.12 Drug prediction

Considering the limitations of existing CRSwNP therapies, potential biomarker-related drugs were predicted using the Drug-Gene Interaction database (DSigDB) (http://dsigdb.tanlab.org/DSigDBv1.0/), and biomarker-drug interaction networks were constructed.

### 2.13 Molecular docking

To refine therapeutic targeting, molecular docking was performed between biomarkers and candidate drugs. Protein structures were obtained from the PDB database (https://www.rcsb.org/), and ligand structures were retrieved from PubChem. Files in PDB format were converted to PDBQT format using AutoDockTools, and docking was conducted using AutoDock Vina. Docking scores of ≤ −5 kcal/mol indicated strong binding affinities, suggesting viable drug-target pairs.

### 2.14 Reverse transcription-quantitative polymerase chain reaction (RT-qPCR)

For RT-qPCR, 10 pairs of CRSwNP and control samples were collected from Sichuan Provincial People’s Hospital. The demographic characteristics of the participants are provided in Supplementary Table S1. All participants provided informed consent, and the study was approved by the ethics committee of Sichuan Provincial People’s Hospital (NO. 2024-4 and date of approval:2023-12-20).

Total RNA was extracted from the 20 samples using TRIzol reagent (Invitrogen, China) following the manufacturer’s protocol, and RNA concentrations were measured with a NanoPhotometer N50. cDNA was synthesized using the SureScript First-Strand cDNA Synthesis Kit (Servicebio, China). qPCR was performed on a CFX Connect Thermal Cycler (Bio-Rad, United States), and mRNA levels were quantified using the 2^−ΔΔCT^ method. The sequences of all primers are detailed in Table 1.

**TABLE 1:** Primer sequence lists.

Primer	Sequences
PRB3 F	CCAGAGCCTCCAGCAAGATG
PRB3 R	GGAGATTCTTCCTGGCTGACA
KRT16 F	CCTATTCTTCCCGCGAGGTC
KRT16 R	GGGAGATAGCTGGGAACTGC
SPAG4 F	TGGGTCTCCAGTAGTCTCTGA
SPAG4 R	ACAGGAAGCGGATGGAACAG
FGFBP1 F	AGGGAGCACATCAAAGGCAA
FGFBP1 R	CGTGTCCTGCACTATGCTGA
GAPDH F	CGAAGGTGGAGTCAACGGATTT
GAPDH R	ATGGGTGGAATCATATTGGAAC

### 2.15 Ethics approval statement

The studies involving human participants were reviewed and approved by the [Sichuan Provincial People’s Hospital’s ethics committee (NO. 2024-4 and date of approval:2023-12-20)]. The patients/participants provided their written informed consent to participate in this study.

### 2.16 Statistical analysis

Statistical analyses were conducted using R software (v4.2.2). Differences between groups were assessed using the Wilcoxon test and t-test. Statistical significance was defined as follows: *P‐value <0.05; **P‐value <0.01; ***P‐value <0.0005; and ****P‐value <0.00005. For GSEA, the criteria were set at |NES| > 1 and p. adjust <0.05.

## 3 Results

### 3.1 Identification of 15 candidate genes

**FIGURE 1:**
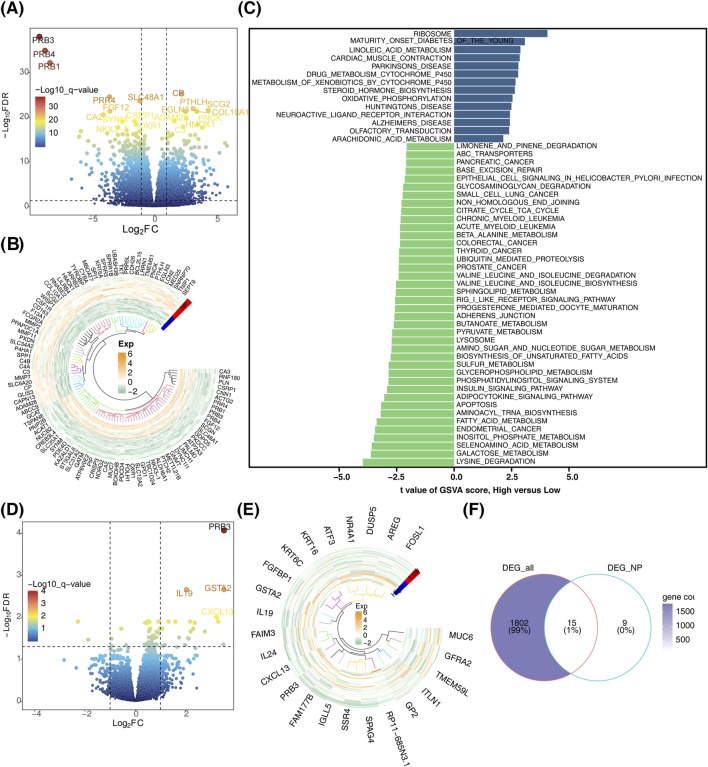
Identification of candidate genes. **(A)** Volcano plot depicting differentially expressed genes. **(B)** Heatmap illustrating the expression patterns of differentially expressed genes. **(C)** Bar chart showing differential gene expression between high and low score subgroups. **(D)** Volcano plot comparing differentially expressed genes between high and low score subgroups. **(E)** Heatmap of differentially expressed genes between high and low score subgroups. **(F)** Venn diagram for the identification of candidate genes.

In this study, a total of 1817 DEGs (DEG_all) were identified between the CRSwNP and control groups, comprising 744 downregulated and 1,073 upregulated genes (Figures 1A, B). Disease group samples were stratified into high- and low-score groups based on the median GSVA score. Distinct pathway activations were observed between these groups: high-score pathways were enriched for “ribosome” and “maturity-onset diabetes of the young,” while low-score pathways included “lysine degradation” and “galactose metabolism” (Figure 1C). Furthermore, 24 DEGs (DEG_NP) were identified between the high- and low-score groups in CRSwNP, comprising 21 upregulated and 3 downregulated genes (Figures 1D, E). By intersecting DEG_all and DEG_NP, 15 candidate genes were identified (Figure 1F).

### 3.2 Enrichment analysis of 15 candidate genes

**FIGURE 2:**
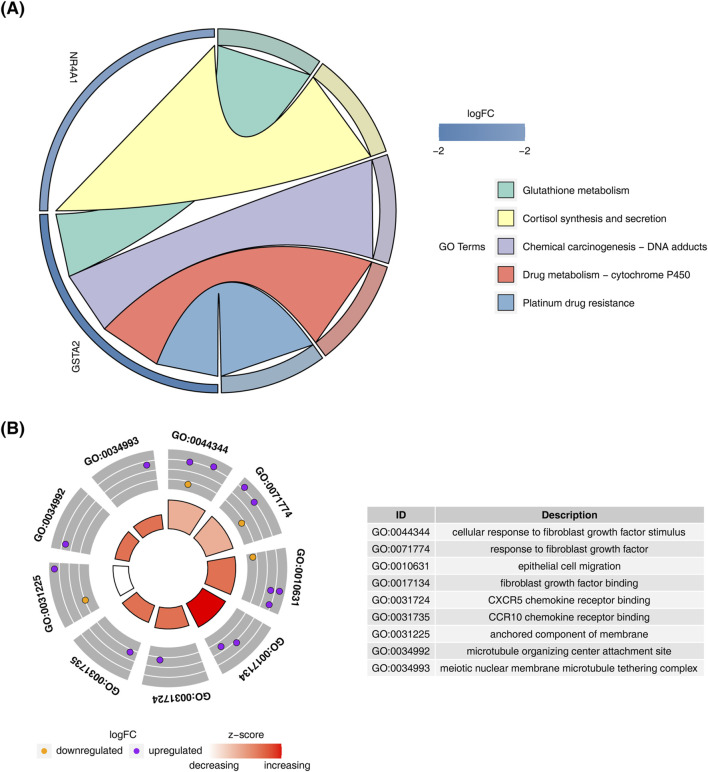
Enrichment analysis of candidate genes. **(A)** KEGG pathway enrichment analysis results for candidate genes. **(B)** GO enrichment analysis results for candidate genes.

KEGG pathway analysis of the candidate genes revealed enrichment in “cortisol synthesis and secretion,” “glutathione metabolism,” and “drug metabolism-other enzymes” (Figure 2A). GO enrichment analysis highlighted processes such as “cellular response to fibroblast growth factor stimulus,” “response to fibroblast growth factor,” and “epithelial cell migration” (Figure 2B).

### 3.3 Identification and validation of 4 biomarkers

**FIGURE 3:**
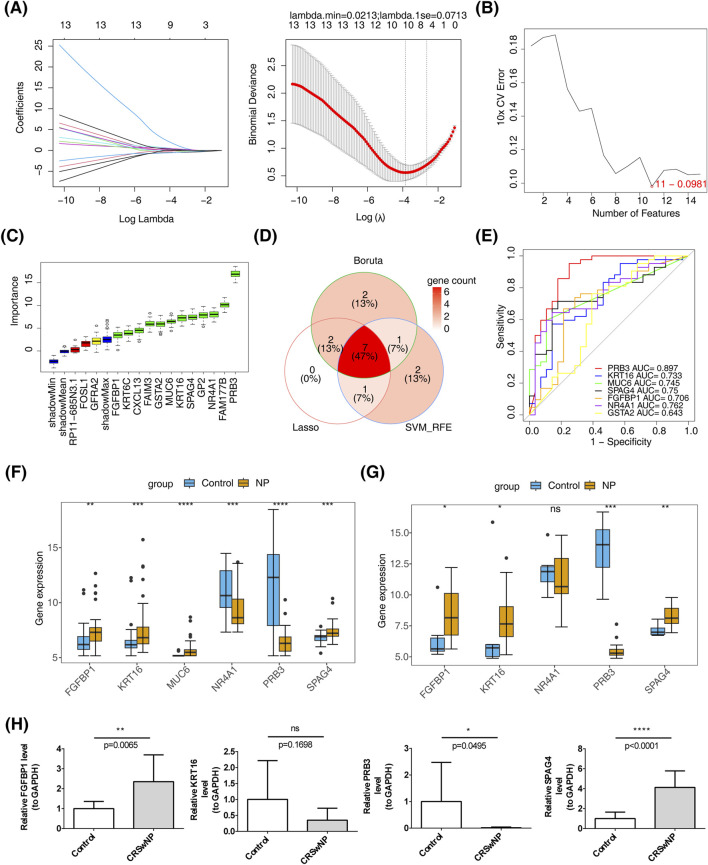
Identification of biomarkers. **(A)** LASSO regression analysis for screening signature genes. **(B)** Plot showing generalization error *versus* the number of features. **(C)** Signature gene selection via the Boruta algorithm. **(D)** Machine learning-based identification of candidate biomarkers. **(E)** Diagnostic value assessment of candidate biomarkers. **(F)** Biomarker expression in the training set GSE136825. **(G)** Biomarker expression in the validation set GSE179265. **(H)** RT-qPCR validation of biomarkers.

Feature gene selection using machine learning methods yielded the following results: LASSO regression identified 10 feature genes—PRB3, KRT16, FAM177B, MUC6, SPAG4, CXCL13, FGFBP1, NR4A1, GSTA2, and GFRA2—at a minimum lambda of 0.0213, where the residual sum of squares was minimized (Figure 3A). SVM-RFE selected 11 feature genes, including MUC6, PRB3, FGFBP1, GSTA2, FOSL1, KRT16, SPAG4, GP2, NR4A1, RP11.685N3.1, and GFRA2, when the error was smallest (Figure 3B). The Boruta algorithm, with a cutoff value of 2.512, identified 12 feature genes: PRB3, KRT16, FAIM3, FAM177B, KRT6C, MUC6, SPAG4, CXCL13, FGFBP1, NR4A1, GP2, and GSTA2 (Figure 3C). By intersecting the results of these machine learning approaches, seven candidate biomarkers were identified: PRB3, KRT16, MUC6, SPAG4, FGFBP1, NR4A1, and GSTA2 (Figure 3D). ROC curve analysis demonstrated that all candidate biomarkers, except GSTA2 (AUC = 0.643), had AUC values greater than 0.7, indicating robust diagnostic value (Figure 3E). Among these, biomarkers with consistent inter-group expression trends were selected. Four genes showed consistent trends across training and validation sets: FGFBP1, KRT16, and SPAG4 were significantly upregulated in the disease group, while PRB3 was notably downregulated (Figures 3F, G). These findings were further validated by RT-qPCR, which confirmed the upregulation of FGFBP1 and SPAG4 and the downregulation of PRB3 in the CRSwNP group (Figure 3H).

### 3.4 Assessment of the risk of CRSwNP based on biomarkers

**FIGURE 4:**
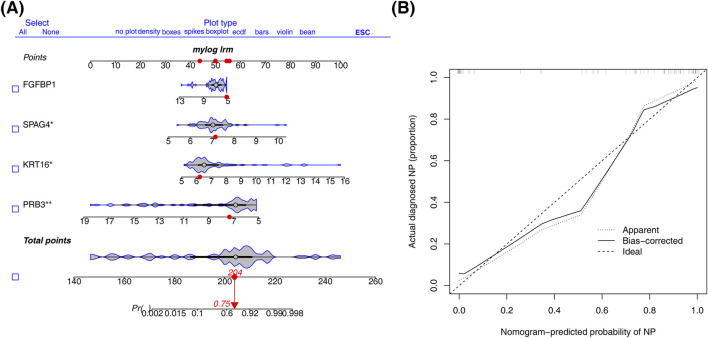
Construction of nomogram. **(A)** Column line diagram based on biomarker construction. **(B)** Calibration curves to assess the predictive accuracy of the nomogram model.

A nomogram was constructed using the four biomarkers (PRB3, KRT16, SPAG4, FGFBP1) to predict the risk of CRSwNP. The total risk score was calculated by summing the individual factor scores (Figure 4A). The predictive accuracy of the nomogram was assessed using a calibration curve, which showed a C-index of 0.957, indicating excellent performance (Figure 4B).

### 3.5 Functional enrichment analysis of biomarkers

**FIGURE 5:**
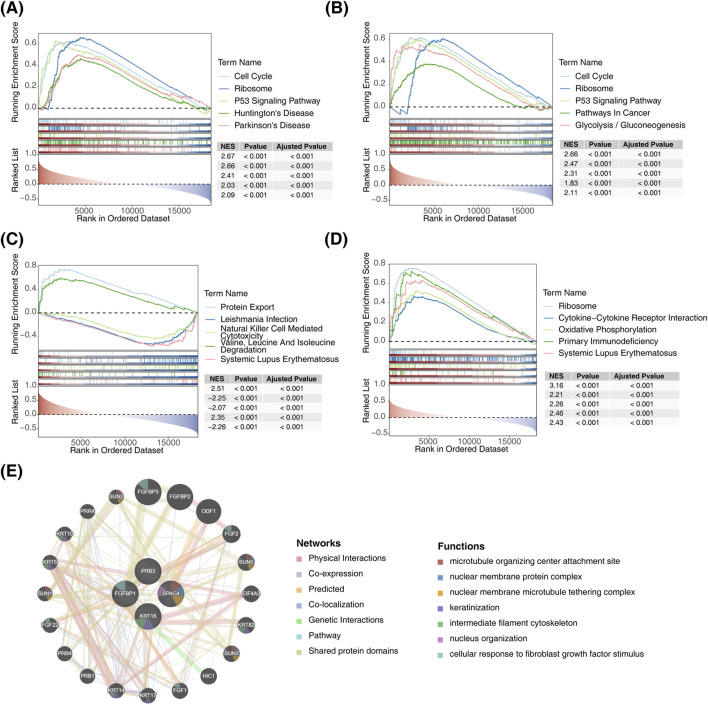
Functional enrichment analysis of biomarkers. **(A)** GSEA of FGFBP1. **(B)** GSEA of KRT16. **(C)** GSEA of PRB3. **(D)** GSEA of SPAG4. **(E)** Interaction network diagram of biomarkers.

According to ssGSEA analysis, the biomarkers exhibited pathway-specific enrichment patterns: FGFBP1 was primarily enriched in the P53 signaling pathway (Figure 5A), KRT16 was enriched in the glycolysis disease-related pathway (Figure 5B), and PRB3 showed enrichment in the protein export pathway (Figure 5C). Additionally, SPAG4 was predominantly linked to the systemic lupus erythematosus disease-related pathway (Figure 5D). Notably, FGFBP1, SPAG4, and KRT16 were collectively enriched in ribosome-associated pathways. To explore biomarker interactions, GeneMANIA was used to integrate data on physical interactions, co-expression, predictions, co-localization, genetic interactions, pathways, and shared protein domains (Figure 5E).

### 3.6 Data controlization and dimensionality reduction

Through data normalization, a total of 18,576 genes and 17,702 cells were obtained (Supplementary Figure S1A, B). To streamline computations, the vst method was applied to extract the top 2000 genes with the highest inter-cell variation coefficients, which were subjected to further analysis (Supplementary Figure S2). PCA showed that cells from different samples were well-mixed without distinct clumps or abnormalities (Supplementary Figure S3). The first 30 principal components, capturing significant variance, were selected for subsequent clustering (Supplementary Figure S4).

### 3.7 Annotation of seven cell types and expression of biomarkers in different cells

**FIGURE 6:**
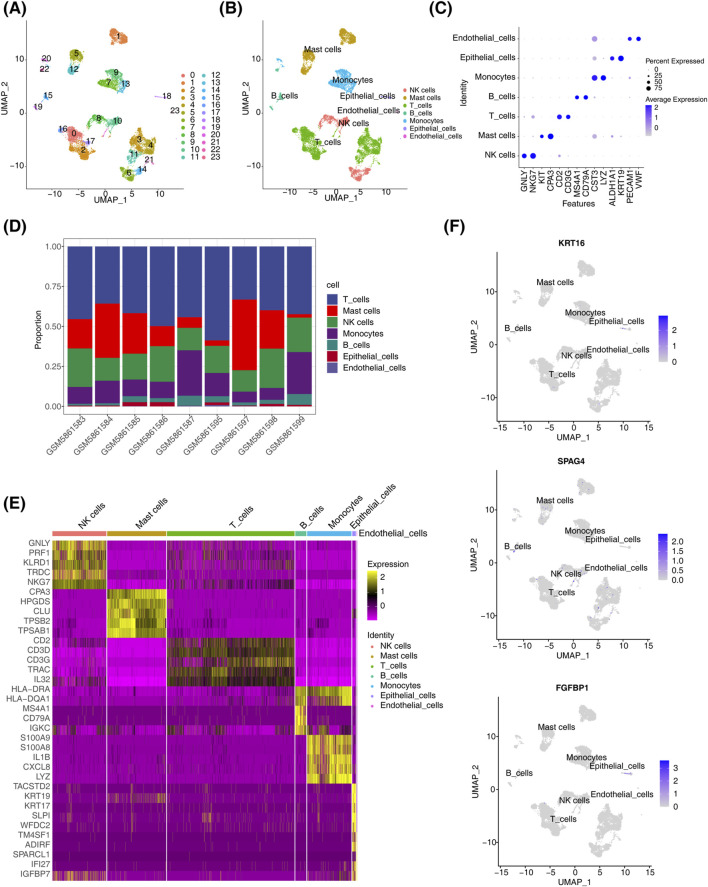
Single-cell analysis results. **(A)** t-SNE distribution map for different cluster samples. **(B)** t-SNE distribution map for samples from different cell types. **(C)** Biomarker expression distribution map. **(D)** Distribution of cell types in CRSwNP. **(E)** Heatmap of differential gene expression in different cell clusters in CRSwNP samples. **(F)** Biomarker expression in different cell clusters.

In dataset GSE196169, all cells were grouped into 24 subtypes (Figure 6A). Based on annotations using the singleR tool and the CellMarker database, seven major cell types were identified: endothelial cells, epithelial cells, monocytes, B cells, T cells, mast cells, and NK cells (Figure 6B). Marker gene analysis revealed that CST3 and LYZ were highly expressed in monocytes, while GNLY and NKG7 were highly expressed in NK cells (Figure 6C). The proportional distribution of different cell types in CRSwNP samples indicated that T cells constituted the largest proportion, whereas epithelial cells represented the smallest (Figure 6D). Using the FindMarker function, DEGs in each cell cluster were identified, and the top five genes for each subpopulation were visualized in a heatmap (Figure 6E). Biomarker expression was further analyzed across different cell clusters. Results showed that KRT16 and FGFBP1 were predominantly expressed in epithelial cells, SPAG4 was primarily expressed in immune cells, while PRB3 expression was not detectable in any specific cell type (Figure 6F).

### 3.8 Regulatory network of biomarkers

**FIGURE 7:**
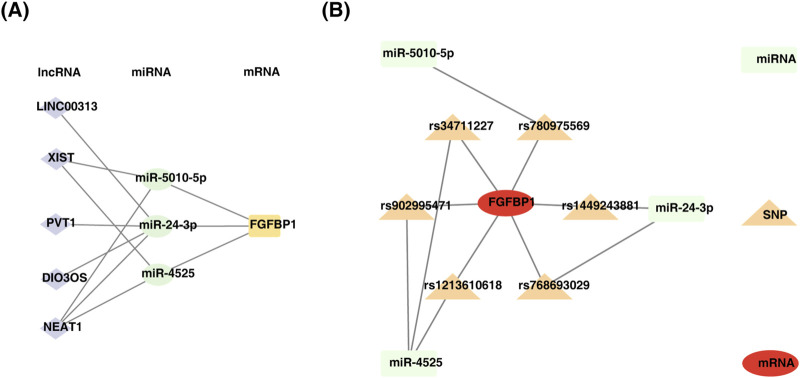
Construction of regulatory networks. **(A)** Diagram of mRNA-miRNA-lncRNA regulatory network. **(B)** Construction of miRNA-SNP-biomarker interaction network.

This study identified 24 miRNAs from the StarBase database and 111 miRNAs from the miRWalk database. By intersecting the results, three miRNAs (miR-5010-5p, miR-24-3p, and miR-4525) were identified. Additionally, five lncRNAs (LINC00313, XIST, PVT1, DIO3OS, and NEAT1) associated with these miRNAs were predicted using StarBase and miRcode. A ceRNA network was constructed, comprising 9 nodes and 11 edges, including interactions such as FGFBP1-miR-5010-5p, FGFBP1-miR-4525, and miR-24-3p-LINC00313 (Figure 7A). Furthermore, six SNPs related to FGFBP1 and its associated miRNAs were predicted (Table 2), and a miRNA-SNP-mRNA network was constructed. This network included 10 nodes (3 miRNAs, 6 SNPs, and 1 mRNA) and 12 edges (6 mRNA-SNP and 6 miRNA-SNP interactions) (Figure 7B). Notably, FGFBP1 was the only biomarker with a complete lncRNA-miRNA-mRNA structure in the ceRNA network.

**TABLE 2:** SNPs for mRNA and miRNA matching.

Gene	SNP	miRNA (loss)
FGFBP1	rs1449243881	miR-24-3p
FGFBP1	rs768693029	miR-24-3p
FGFBP1	rs1213610618	miR-4525
FGFBP1	rs902995471	miR-4525
FGFBP1	rs34711227	miR-4525
FGFBP1	rs780975569	miR-5010-5p

### 3.9 Biomarker distribution in different cells

**FIGURE 8:**
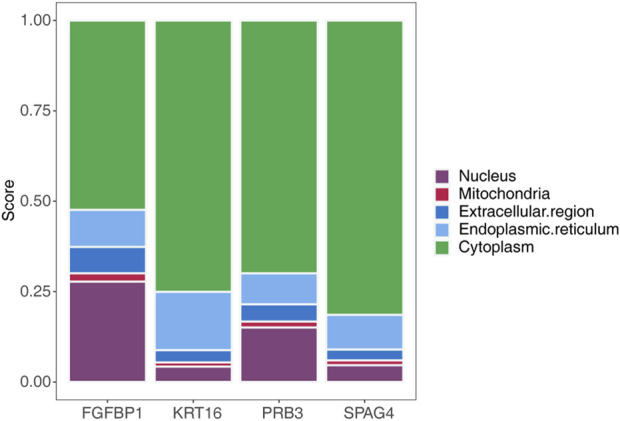
Subcellular localization analysis map of biomarkers.

Subcellular localization analysis of the biomarkers using mRNALocater revealed their distribution across various subcellular compartments, with the highest expression levels observed in the cytoplasm and the lowest in mitochondria (Figure 8). The localization sequences for the biomarkers were as follows: FGFBP1: cytoplasm > nucleus > endoplasmic reticulum > extracellular region > mitochondria; KRT16: cytoplasm > endoplasmic reticulum > nucleus > extracellular region > mitochondria; PRB3: cytoplasm > nucleus > endoplasmic reticulum > extracellular region > mitochondria; SPAG4: cytoplasm > endoplasmic reticulum > nucleus > extracellular region > mitochondria.

### 3.10 Biomarkers-related drugs were obtained

**FIGURE 9:**
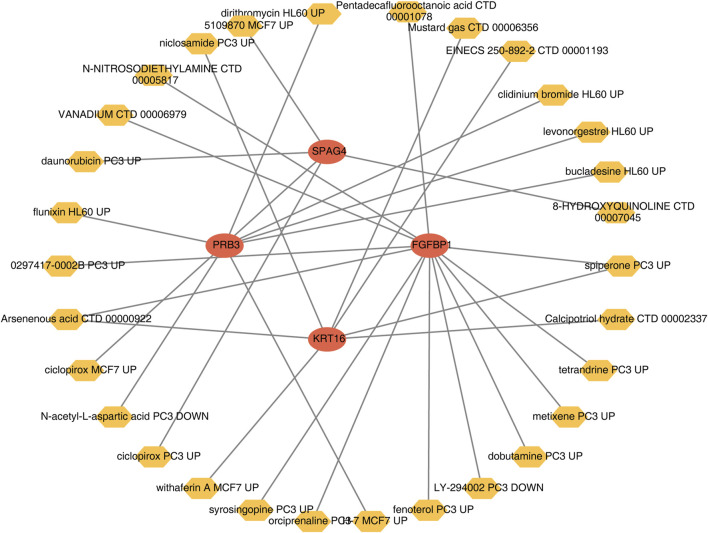
Biomarker-drug regulatory network.

A wide range of drugs associated with the biomarkers was identified, including spiperone PC3 UP, H-7 MCF7 UP, and syrosingopine PC3 UP (Table 3). A biomarker-drug network was constructed, comprising 34 nodes and 32 edges (Figure 9). Examples of interactions in this network include FGFBP1-orciprenaline PC3 UP, PRB3-H-7 MCF7 UP, and KRT16-calcipotriol hydrate CTD 00002337.

**TABLE 3:** Drugs associated with biomarkers.

Gene	Drug
FGFBP1	spiperone PC3 UP
KRT16	spiperone PC3 UP
KRT16	Calcipotriol hydrate CTD 00002337
FGFBP1	tetrandrine PC3 UP
FGFBP1	metixene PC3 UP
FGFBP1	dobutamine PC3 UP
FGFBP1	LY-294002 PC3 DOWN
FGFBP1	fenoterol PC3 UP
PRB3	H-7 MCF7 UP
FGFBP1	orciprenaline PC3 UP
FGFBP1	syrosingopine PC3 UP
KRT16	withaferin A MCF7 UP
SPAG4	ciclopirox PC3 UP
PRB3	N-acetyl-L-aspartic acid PC3 DOWN
SPAG4	ciclopirox MCF7 UP
KRT16	Arsenous acid CTD 00000922
FGFBP1	Arsenous acid CTD 00000922
FGFBP1	0297417-0002B PC3 UP
PRB3	flunixin HL60 UP
SPAG4	daunorubicin PC3 UP
FGFBP1	VANADIUM CTD 00006979
FGFBP1	N-NITROSODIETHYLAMINE CTD 00005817
KRT16	niclosamide PC3 UP
SPAG4	5109870 MCF7 UP
PRB3	dirithromycin HL60 UP
FGFBP1	Pentadecafluorooctanoic acid CTD 00001078
KRT16	Mustard gas CTD 00006356
KRT16	EINECS 250-892-2 CTD 00001193
PRB3	clidinium bromide HL60 UP
PRB3	levonorgestrel HL60 UP
PRB3	bucladesine HL60 UP
SPAG4	8-HYDROXYQUINOLINE CTD 00007045

### 3.11 Molecular docking

**FIGURE 10:**
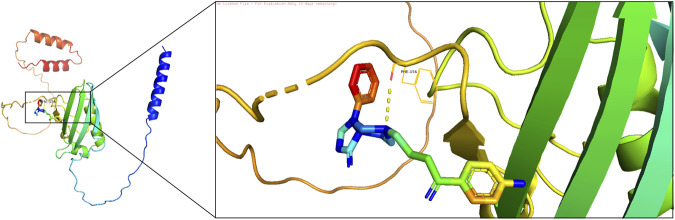
Docking interaction diagram between spiperone and FGFBP1.

Molecular docking was performed to evaluate the effectiveness of newly predicted drugs. Docking results were visualized using PyMol, and the binding energy between protein receptors and small molecule ligands was used to assess binding activity. Lower binding energy indicated stronger binding affinity and stability. A docking score of ≤ −5 kcal/mol was considered indicative of strong compound-target binding affinity, suggesting potential therapeutic targets. Among the predicted drugs, spiperone and arsenous acid were selected for further evaluation. The binding energy for the interaction between spiperone and FGFBP1 was −6.63 kcal/mol, indicating a strong binding affinity (Figure 10), supporting its potential as a therapeutic compound for CRSwNP.

## 4 Discussion

CRS affects over 10% of the adult population in Europe and the United States, while its prevalence ranges from 5% to 10% among adults in Asia (Bachert et al., 2020). CRSwNP is a distinct phenotype of CRS, characterized by a significant disease burden and symptoms such as facial pain and anosmia (Stevens et al., 2016). The etiology of nasal polyps has been attributed to factors like pseudocyst formation, edema, and structural or functional alterations in the submucosal glands (Takabayashi et al., 2013). Recent studies suggest that epithelial dysfunction, type 2 inflammation, and fibrin deposition may play a pivotal role in polyp development (Takabayashi et al., 2013). Notably, approximately 85% of patients with CRSwNP exhibit eosinophilic or type 2 inflammation (Bachert et al., 2021), marked by elevated levels of interleukins (IL) such as IL-4, IL-5, IL-9, IL-13, IL-25, and IL-33 (Bachert and Akdis, 2016). The roles of the IL-10 family of cytokines in CRSwNP have recently garnered increasing attention due to their involvement in the diagnosis and treatment of autoimmune diseases, cancer, and inflammatory disorders (Calimeri et al., 2024; Salkeni and Naing, 2023; Kotlarz et al., 2012). However, their specific biological functions in CRSwNP remain underexplored. In this study, bioinformatics methods were used to identify four IL-10 family-related biomarkers and novel drug candidates for CRSwNP treatment. Potential therapeutic targets for spiperone and arsenous acid were also identified via molecular docking, providing a theoretical basis for future clinical research.

The results demonstrated that FGFBP1, KRT16, and SPAG4 were significantly upregulated in CRSwNP samples, whereas PRB3 was markedly downregulated. Analysis of the biomarkers revealed that all had AUC values exceeding 0.7, indicating their diagnostic utility. An AUC greater than 0.7 generally reflects good discriminatory capacity (Davies et al., 2023). The selection of this threshold was based on the characteristics of the study samples and the diagnostic requirements of CRSwNP. Comparisons with other studies revealed that many similarly employed an AUC >0.7 as a valid biomarker criterion (Wang H. et al., 2024), while some adopted a stricter threshold of AUC ≥0.8 for higher diagnostic performance. Nevertheless, in this study, an AUC >0.7 was sufficient to demonstrate clinical significance. Notably, the application of these four IL-10 family-related biomarkers in CRSwNP has not been previously reported.

Poly-L-arginine has been shown to stimulate angiogenesis in asthma by inducing the expression of Fibroblast Growth Factor Binding Protein 1 (FGFBP1) in epithelial cells. This effect is mediated through the activation of the mTORC1-STAT3 signaling pathway, positioning poly-L-arginine as a potential therapeutic target for asthma treatment (Chen et al., 2021). A recent study published in Cell identified a novel population of regenerative stem cells expressing FGFBP1 within the upper intestinal epithelial recesses. These cells, distinct from Lgr5+ cells, were demonstrated through time-resolved fate mapping and lineage tracing to generate Lgr5+ basal columnar cells and other intestinal lineages. Moreover, these FGFBP1-expressing stem cells persisted in intestinal epithelial regeneration following Lgr5+ cell depletion (Capdevila et al., 2024). Conditional knockout experiments in mice further established that FGFBP1 expression in upper crypt stem cells is critical for crypt regeneration and maintaining intestinal epithelial homeostasis (Capdevila et al., 2024). The 2012 European Position Paper on Rhinosinusitis and Nasal Polyps highlighted abnormal epithelial remodeling and chronic inflammation as key pathological features of CRSwNP (Fokkens et al., 2012). By analogy, FGFBP1 is hypothesized to play a significant role in the dysregulated remodeling of nasal epithelium in CRSwNP. Analysis using ssGSEA revealed that FGFBP1 is predominantly enriched in the p53 signaling pathway. Experimental studies on exosomes derived from nasal lavage fluid and mucosal epithelial cells of patients with CRSwNP and healthy controls indicated that exosomes from impaired epithelial tissues contain differentially expressed proteins primarily associated with epithelial remodeling via the p53 signaling pathway (Zhou M. et al., 2020). Additionally, analysis of the ceRNA network identified FGFBP1 as the only biomarker with a complete lncRNA-miRNA-mRNA regulatory structure, participating in multiple interactions as a central node. These findings underscore the pivotal roles of FGFBP1 and the p53 signaling pathway in the pathogenesis of CRSwNP.

KRT16 has been significantly upregulated in metastatic lung cancer tissues and identified as a prognostic marker associated with poor overall survival (Wang et al., 2023). Mechanistic studies using transwell assays and xenograft mouse models demonstrated that KRT16 knockdown reduces lung cancer metastasis in both *in vitro* and *in vivo* settings (Wang et al., 2023). In the context of CRSwNP, KRT16 is implicated in epithelial cell proliferation, differentiation, and repair, processes that parallel epithelial dysfunction, including compromised barrier integrity. Its role in CRSwNP may involve modulating chronic inflammation in the nasal cavity and sinuses through effects on epithelial stress responses, repair mechanisms, or immune modulation.

Further research demonstrated that KRT16 knockdown significantly affects LUAD cell migration, invasion, proliferation, and EMT. TFAP2A was identified as a transcriptional regulator driving KRT16 overexpression and enhancing its tumorigenic potential. High KRT16 levels were associated with poor prognosis in patients with LUAD, establishing it as an independent prognostic marker (Nicholson, 1988). Additionally, studies on skin barrier disorders revealed that KRT6, KRT16, and KRT17 serve as early indicators of barrier damage. Elevated expression of these proteins disrupts cell proliferation, adhesion, migration, and inflammatory balance in keratinocytes, leading to excessive growth and immune activation. This cascade triggers autoimmune responses driving the development of psoriasis (Zhang et al., 2019).

According to ssGSEA, KRT16 was enriched in the glycolysis disease-related pathway in CRSwNP. Single-cell RNA sequencing studies in CRS have demonstrated increased expression of genes encoding glycolytic enzymes in epithelial cells, stromal cells, and memory T-cell subsets in patients with CRSwNP compared to healthy controls, highlighting the critical role of glycolytic reprogramming in tissue remodeling (Huang et al., 2024). Glycolysis, a fundamental pathway for cellular energy metabolism, likely influences epithelial cell proliferation, immune responses, and inflammatory processes in nasal polyps. The aberrant metabolic state observed in CRSwNP, including enhanced glycolysis, may contribute to epithelial dysfunction and chronic inflammation, thereby promoting nasal polyp formation. In this context, KRT16 is proposed to regulate cell proliferation, repair, and immune responses via glycolysis, closely linking it to the tissue remodeling processes characteristic of nasal polyps. Although direct evidence connecting KRT16 to CRSwNP remains unavailable, further investigation is warranted to clarify its role as a potential therapeutic target. Future research will focus on elucidating the molecular mechanisms underlying KRT16’s involvement in CRSwNP to enhance both understanding and therapeutic interventions.

Database analysis and immunohistochemistry have revealed elevated SPAG4 levels with prognostic significance in liver cancer. RNA sequencing studies indicate that SPAG4 overexpression activates the lipogenesis state and the SREBP1-mediated pathway, providing evidence for its role in lipid metabolism dysregulation and tumor progression in hepatocellular carcinoma (Liu et al., 2022). Analogously, recent studies on CRS, using animal models, *in vitro* human cell cultures, and dietary analyses, suggest that significant alterations in lipid mediator signaling are involved in the disease’s pathophysiology (Robinson et al., 2023). In CRSwNP, SPAG4 may influence biological signal transduction through its impact on lipid metabolism, potentially contributing to disease progression. Furthermore, SPAG4 upregulation in renal clear cell carcinoma (RCC), regulated by hypoxia via HIF-1 and VHL, has been shown to enhance tumor cell migration and invasion, while SPAG4 knockdown reduces RCC cell invasiveness *in vitro* (Knaup et al., 2014). In CRSwNP, the upregulation of SPAG4 might similarly play a role in the invasion of the sinus wall, positioning it as a compelling target for clinical intervention.

A study of prolactinomas using exon gene sequencing and RT-qPCR quantitative analysis revealed that PRB3 mRNA levels were approximately four times lower in drug-resistant prolactinomas compared to responsive tumors (*p* = 0.02). Additionally, reduced PRB3 expression was associated with tumor recurrence, suggesting that low PRB3 mRNA levels may contribute to dopamine agonist resistance and tumor recurrence in prolactinomas (Wang et al., 2014). Similarly, the present study identified significant downregulation of PRB3 in CRSwNP compared to the control group, prompting the hypothesis that it may play a role in the recurrence of CRSwNP. ssGSEA analysis further indicated that PRB3 was enriched in the natural killer (NK) cell-mediated cytotoxicity pathway. NK cells, multifaceted lymphocytes of the innate immune system, are essential for executing key functions in host defense and immune regulation. Impaired NK cell function in individuals with CRS has been correlated with poor prognostic outcomes, potentially contributing to the development of asthma. Moreover, NK cells are integral to regulating both the initiation and progression of CRS, underscoring their critical role in maintaining immune homeostasis and mitigating disease severity (Kim et al., 2013). Thus, it is hypothesized that PRB3 may influence NK cell-mediated cytotoxicity, contributing to the pathophysiological progression of CRSwNP. This hypothesis emphasizes the necessity for further investigation into the complex interaction between PRB3 and NK cell function in the context of CRSwNP.

Epithelial-mesenchymal transition (EMT) is a critical cellular process in the pathogenesis of CRSwNP, where epithelial cells play a central role (Zhou X. et al., 2020). Apical epithelial cells, located at the luminal surface, are crucial for maintaining the integrity of the epithelial barrier. A reduction in these cells signifies compromised barrier function in nasal polyps and peripolyposis tissues, leading to increased epithelial permeability and facilitating the transmigration of microbial agents and antigens. This enhanced permeability triggers an inflammatory cascade that exacerbates CRSwNP (Wang Y. et al., 2024). Additionally, colonization by Aspergillus flavus has been identified as a key initiator of nasal polypogenesis, recruiting T cells to the nasal mucosa. This recruitment not only accelerates nasal polyp progression but also contributes to the inflammatory environment characteristic of CRSwNP (Rai et al., 2023). In the present study, biomarker expression was meticulously analyzed across discrete cellular clusters. Our findings revealed that KRT16 and FGFBP1, markers of epithelial differentiation, are predominantly expressed in epithelial cells. In contrast, SPAG4, a gene associated with immune cell function, is primarily expressed in immune cell populations. These findings highlight the cellular heterogeneity in CRSwNP and provide a robust framework for understanding the pathobiology of this disease, offering valuable insights for the development of targeted therapeutic strategies.

In this study, numerous drugs associated with biomarkers were identified. Drug-target docking studies were employed to assess the efficacy of newly predicted drugs, with spiperone and arsenous acid selected as promising candidates. Spiperone, an antipsychotic drug, is known to interact with dopamine receptors, particularly the D2 receptor (Im et al., 2020). It has been shown to mediate endothelial regeneration in an animal model of chronic obstructive pulmonary disease (COPD) by enhancing the mobilization and migration of endothelial progenitor cells (EPCs, CD45^−^CD34^+^CD31^+^), CD309+-endothelial cells, and angiogenesis precursors (CD45^−^CD117+CD309+) to the lung (Skurikhin et al., 2021). Given that the nasal passages and lungs are part of the same respiratory tract, spiperone may have a beneficial effect in patients with CRSwNP. Arsenous acid has been reported to suppress the production of pro-inflammatory cytokines and alleviate respiratory tract infections (Islam et al., 2022). In conclusion, spiperone and arsenous acid show potential as therapeutic agents for CRSwNP, though continued exploration of additional therapeutic options is necessary.

In recent years, biologics targeting type 2 inflammation, including IL-4, IL-5, IL-13, and IgE, have demonstrated efficacy in treating severe cases of CRSwNP that are resistant to glucocorticoids and surgical interventions. Despite this advancement, a significant proportion of patients, ranging from 40% to 60%, continue to show insufficient responses to these biologics (Han et al., 2021). Three type 2 biologics—dupilumab, mepolizumab, and omalizumab—have received FDA/EMA approval for the treatment of severe, uncontrolled CRSwNP. Mepolizumab targets IL-5 to inhibit eosinophil activity, dupilumab blocks IL-4 and IL-13 signaling by targeting the IL-4 receptor α subunit, and omalizumab prevents IgE from interacting with mast cell and basophil receptors, thereby inhibiting IgE-mediated allergic responses (Fujieda et al., 2024; Gevaert et al., 2020; Bachert et al., 2019). However, the efficacy of these drugs may vary among individuals, highlighting the need for personalized treatment strategies and continuous monitoring of patient responses.

This study is the first to identify four IL-10 family-related biomarkers in CRSwNP, an important contribution to the diagnosis and treatment of the disease. However, the study has several limitations. In the RT-qPCR validation phase, only 10 pairs of samples were analyzed, resulting in a small sample size. This limited the accuracy of the results and may not fully represent the broader patient population, potentially introducing biases. Future studies should expand the sample size and conduct large-scale cohort studies to validate the findings, thus enhancing the reliability and generalizability of the results.

While molecular docking in the current study provided preliminary insights into the interactions of spiperone and arsenous acid with CRSwNP, the precise relationship between these compounds and the disease pathophysiology remains unclear. Relying solely on molecular docking may not capture the full complexity of their mechanisms in the biological context. Furthermore, there is insufficient data to compare these compounds directly with existing therapeutic agents, which could lead to an incomplete assessment of their relative advantages or disadvantages. Without such comparisons, it is challenging to ascertain whether these compounds hold true therapeutic potential for CRSwNP.

Future research should delve deeper into the specific target sites and signaling pathways of spiperone and arsenous acid in CRSwNP. Various experimental approaches, such as gene knockout or knockdown animal models, can be employed to observe disease progression in the absence of specific targets and to examine the modulation of disease-related signaling pathways by these drug candidates. For instance, CRSwNP mouse models can be developed, specific target genes identified through molecular docking can be knocked out, and the effects of spiperone and arsenous acid on nasal polyp formation, inflammatory cell infiltration, and other disease markers can be studied to better understand their therapeutic potential.

If established ligands for CRSwNP are available, detailed comparative studies of molecular docking binding energies should be conducted. In parallel, it is essential to evaluate not only the binding energies but also the pharmacokinetics, pharmacodynamics, and other relevant drug properties. *In vitro* assays using nasal mucosal epithelial cells or inflammatory cell lines derived from patients with CRSwNP can serve to compare the cellular uptake and inflammatory factor secretion profiles of spiperone, arsenous acid, and existing ligands. Additionally, *in vivo* animal models are crucial to assess the therapeutic potential of these compounds in alleviating CRSwNP symptoms and reducing nasal polyp size, thereby offering a more comprehensive basis for their clinical application.

Multi-omics approaches, encompassing transcriptomics, proteomics, metabolomics, and other techniques, should be employed to investigate the broader effects of spiperone and arsenous acid on CRSwNP. Through multi-omics analysis of pre- and post-treatment tissue or blood samples from patients with CRSwNP, novel biomarkers and mechanisms of action can be identified. Specifically, transcriptomic analysis can elucidate the regulatory effects of these drugs on disease-associated gene expression, proteomics can reveal alterations in protein expression and post-translational modifications induced by the drugs, and metabolomics can provide insights into the impact of the drugs on metabolic profiles. Collectively, these approaches will enhance the understanding of drug-disease interactions from a systems biology perspective.

## Data Availability

The datasets presented in this study can be found in online repositories. The names of the repository/repositories and accession number(s) can be found in the article/Supplementary Material.

## Abbreviations

CRSwNP, chronic rhinosinusitis with nasal polyps; CRS, Chronic rhinosinusitis; CRSsNP, chronic rhinosinusitis without nasal polyps; SVM-RFE, support vector machine recursive feature elimination; DEGs, differentially expressed genes; DEGs_all, differentially expressed genes in GRSwNP and control groups; DEGs_NP, differentially expressed genes between high- and low-scores groups in the GRSwNP group; LASSO, least absolute shrinkage and selection operator; scRNA-seq, single-cell RNA sequencing data; RT-qPCR, reverse transcription-quantitative polymerase chain reaction; PCA, principal component analysis; GEO, gene expression omnibus; GO, gene ontology; KEGG, kyoto encyclopedia of genes and genomes; AUC, area under curve; BP, biological process; MF, molecular function; CC, cellular component; ROC, receiver operating characteristic; GSVA, gene set variation analysis; DECs, differentially infiltrated immune cells; GSEA, gene set enrichment analysis.
