# Supplementary material for: Unveiling and validating biomarkers related to the IL-10 family in chronic sinusitis with nasal polyps: insights from transcriptomics and single-cell RNA sequencing analysis
Source: Front Mol Biosci. 2025 Jan 3;11:1513951. doi: 10.3389/fmolb.2024.1513951 (PMC11738911; doi:10.3389/fmolb.2024.1513951)
Supplement: Supplementary file 1 [file Table1.pdf]

CRSwNP	SEX(MALE/FEMALE)	AGE	sinuses involved in surgery
1	FEMALE	37	Bilateral ethmoid/sphenoid/frontal sinuses
2	FEMALE	69	Bilateral maxillary/ethmoid/frontal/sphenoid sinus
3	FEMALE	53	Bilateral maxillary/ethmoid/frontal/sphenoid sinus
4	MALE	21	Bilateral maxillary/ethmoid/frontal/sphenoid sinus
5	MALE	61	Bilateral maxillary/ethmoid/sphenoid sinuses
6	FEMALE	23	Unilateral maxillary sinus
7	FEMALE	64	Unilateral maxillary sinus
8	FEMALE	58	Bilateral maxillary/ethmoid/frontal/sphenoid sinus
9	MALE	66	Bilateral maxillary/ethmoid/sphenoid sinuses
10	MALE	18	Bilateral maxillary/ethmoidal sinuses
**CONTROLS**	**SEX(MALE/FEMALE)**	**AGE**	**diagnosis**
1’	MALE	35	Deviated septum, allergic rhinitis
2’	MALE	30	Deviated septum, snoring
3’	MALE	28	Deviated septum, allergic rhinitis
4’	FEMALE	56	Deviated septum, allergic rhinitis
5’	MALE	29	Deviated septum, allergic rhinitis
6’	MALE	54	Deviated septum, allergic rhinitis
7’	MALE	37	Deviated septum, allergic rhinitis
8’	MALE	22	Deviated septum
9’	MALE	50	Deviated septum, allergic rhinitis
10’	MALE	50	Deviated septum
