# Supplementary material for: Unveiling and validating biomarkers related to the IL-10 family in chronic sinusitis with nasal polyps: insights from transcriptomics and single-cell RNA sequencing analysis
Source: Front Mol Biosci. 2025 Jan 3;11:1513951. doi: 10.3389/fmolb.2024.1513951 (PMC11738911; doi:10.3389/fmolb.2024.1513951)
Supplement: Supplementary file 2 [file DataSheet1.pdf]

Supplementary Material

1 Supplementary Figures and Tables

1.1 Supplementary Figures

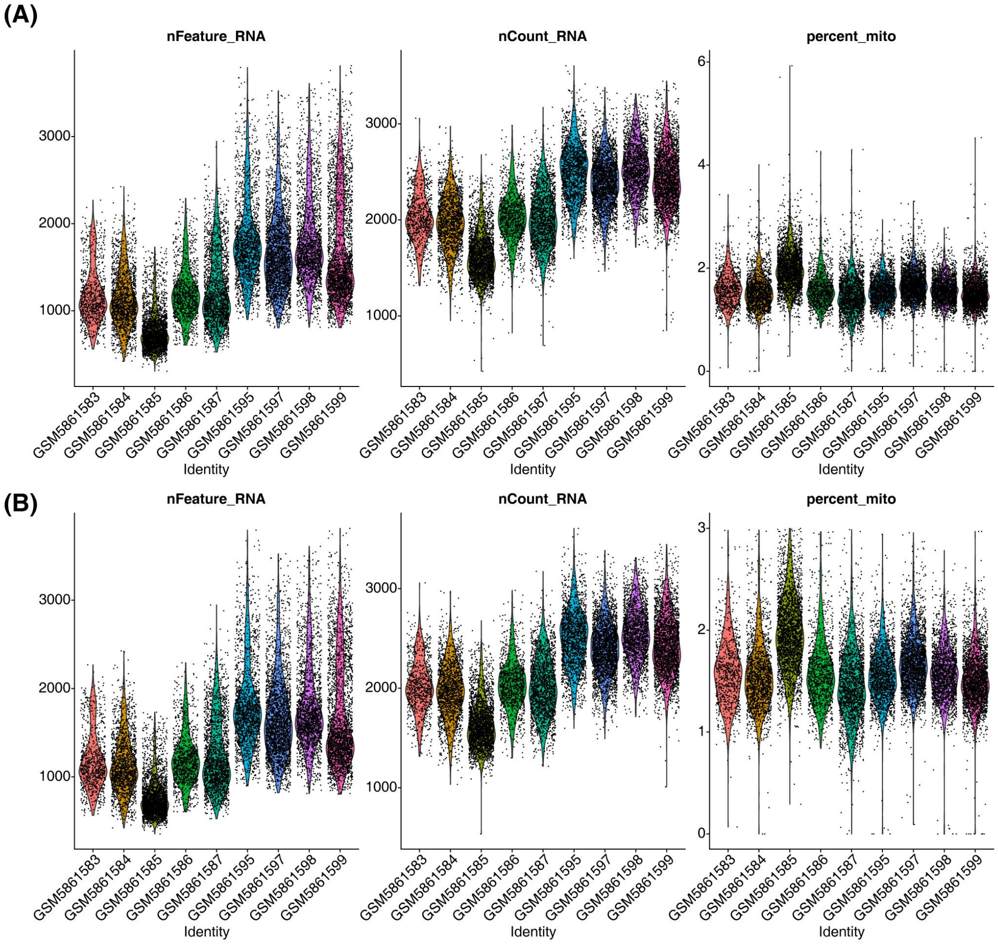

Supplementary Figure 1. Distribution of genes, UMI count content and percentage of mitochondrial gene expression in each cell in the sample.

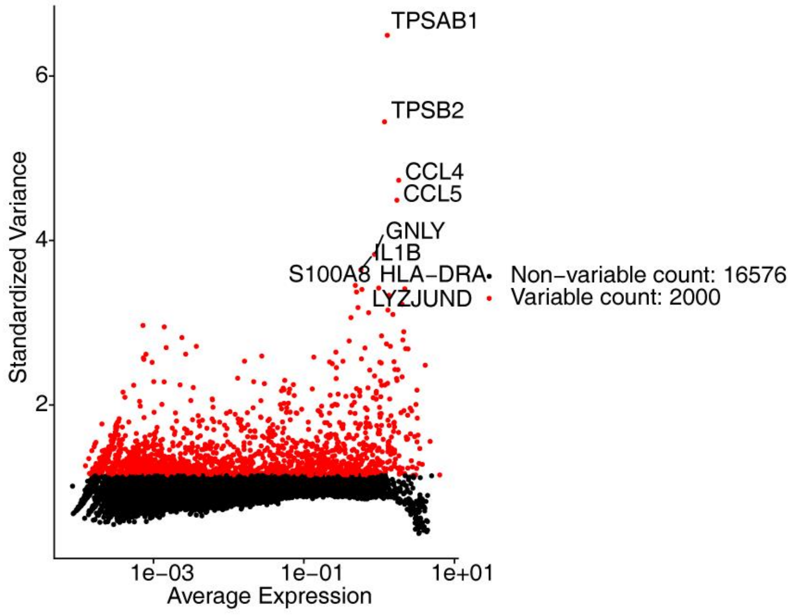

Supplementary Figure 2. Scatterplot of highly variable genes.

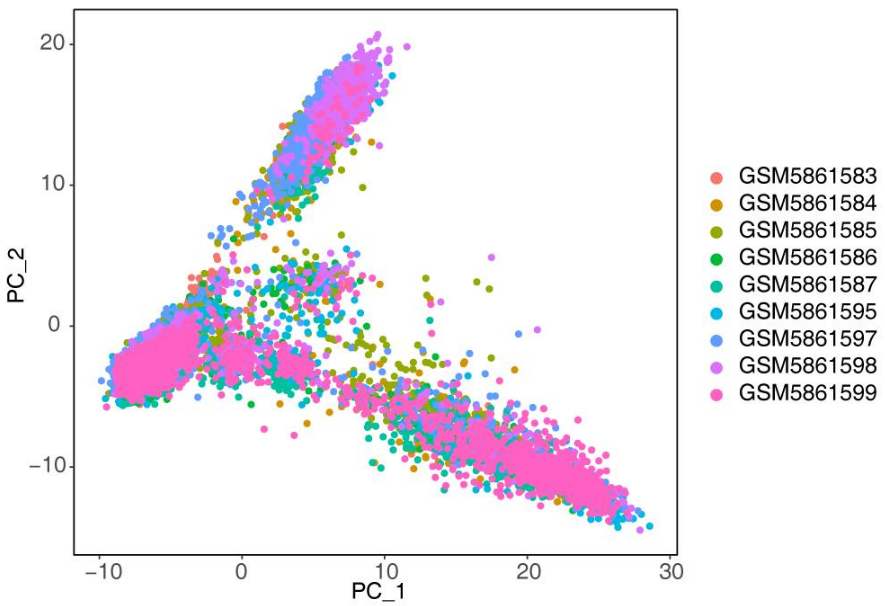

Supplementary Figure 3. Distribution of PCA cells.

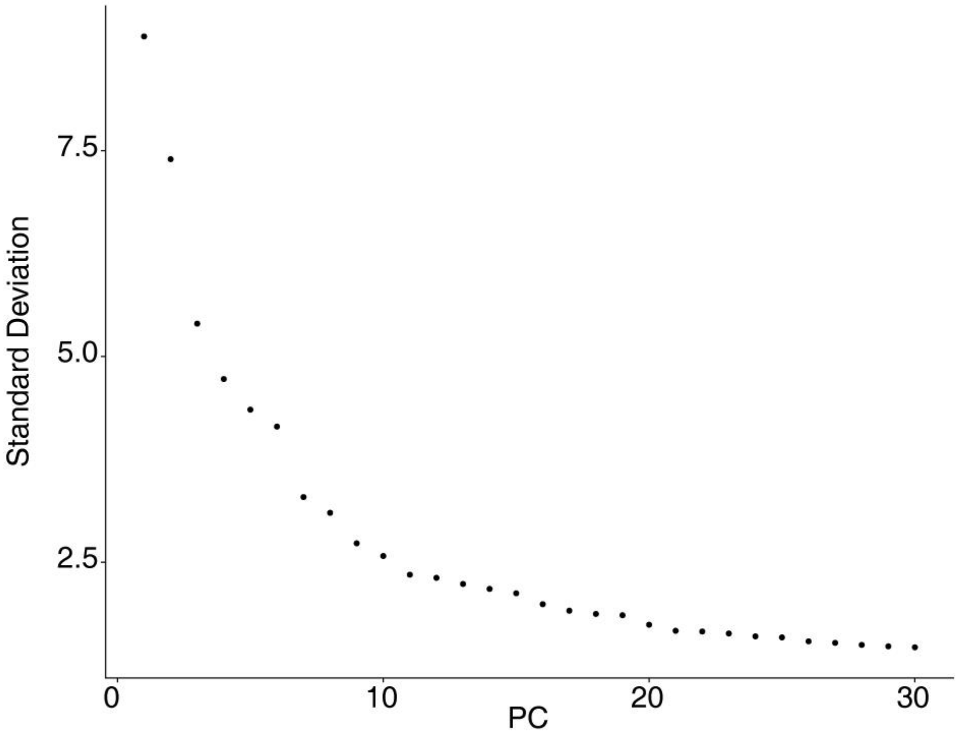

Supplementary Figure 4. PCA inflection point chart.
